# Mechanical Behavior of Bamboo-Like Structures under Transversal Compressive Loading

**DOI:** 10.3390/biomimetics8010103

**Published:** 2023-03-05

**Authors:** Siyi Wang, Jiayang Wang, Kyriakos Komvopoulos

**Affiliations:** Department of Mechanical Engineering, University of California, Berkeley, CA 94720, USA

**Keywords:** bamboo, biomimetic materials, deformation, fracture, mechanical properties, strength, structure architecture, unit cell

## Abstract

Inspired by many biological structures in nature, biomimetic structures demonstrate significantly better mechanical performance than traditional engineering structures. The exceptional mechanical properties of natural materials are attributed to the hierarchical architecture of their structure. Consequently, the implementation of biomimetic structures in the design of lightweight structures with tailored mechanical properties has been constantly increasing in many fields of science and engineering. The bamboo structure is of particular interest because it combines a light weight and excellent mechanical properties, often surpassing those of several engineering materials. The objective of this study was to evaluate the mechanical behavior of bamboo-inspired structures subjected to transversal compressive loading. Structures consisting of bamboo-like thin-walled hexagonal building blocks (unit cells) with different dimensions were fabricated by stereolithography 3D printing and their mechanical performance was evaluated by mechanical testing, high-speed camera video recordings, and finite element simulations. The results of the elastic modulus, yield strength, and strain energy density at fracture were interpreted in terms of characteristic dimensions of the unit cell structure. The failure process was elucidated in the light of images of the fractured structures and simulation strain maps. The results of this study demonstrate that ultralight bamboo-like structures with specific mechanical characteristics can be produced by optimizing the dimensions and number density of the hexagonal unit cell.

## 1. Introduction

Architected structures with different unit cells emulating biomaterial microstructures have captured the attention of the scientific community. Various hierarchical material structures in nature have inspired the design of novel biomimetic structures. The discovery of fabrication approaches for developing structure architectures similar to those of nature materials is referred to as biomimicry. Foregoing difficulties in fabricating biomimetic structures were overcome as a result of advances in additive manufacturing or three-dimensional (3D) printing technologies, enabling the development of complex structures with exceptional mechanical properties.

Bioinspired structures resembling strong and tough biomaterials (e.g., tooth enamel, bamboo, nacre, toucan beak, and walnut) or structures with unit cells (building blocks) that mimic the lattice of biomaterials promise to surpass the conflict of strength versus toughness of traditional materials [1]. For example, various biomimetic structures demonstrate significantly better energy absorption capabilities than monolithic structures with the same composition. This is attributed to the hierarchical architecture of these structures, consisting of unit cells with a well-defined size, shape, and arrangement that demonstrate different levels of organization [2]. Consequently, major advances have been made in the design of biomimetic, lightweight structures displaying an excellent energy absorption capacity [3].

Biomaterials can be grouped into several categories, depending on their structure architecture and lattice (unit cell) geometry. Layered structures comprise alternating layers, resulting in both high stiffness and toughness. A representative example from nature is nacre, which consists of hexagonal microplatelets of aragonite arranged in continuous parallel laminae and separated by sheets of an organic matrix composed of elastic biopoly- mers such as chitin, lustrin, and silk-like proteins. The inelastic deformation of nacre under shear occurs by interlamellae slip and under tension through the development of multiple dilatation bands at intertablet boundaries accompanied by interlamellae sliding [4]. The excellent mechanical properties of nacre are attributed to its hierarchical structure and organic/inorganic interfaces. The combination of brittle platelets and thin layers of elastic biopolymers makes this material strong and resilient. In view of that, the hierarchical structure of nacre was imitated in the design and fabrication of multilayer composite laminates assembled from 3D polygonal tablets that were bonded with organic adhesives [5]. Nacre-inspired laminated composite structures demonstrate remarkable toughness, good impact resistance, and high strength [6,7,8]. The toughness of synthetic nacre-like composites can be enhanced by increasing the volume fraction of the organic matrix (at the expense of a lower yield strength), or by decreasing the elastic modulus of the organic matrix for a certain volume fraction of the organic matrix [9].

Another important classification of lightweight biomimetic structures exhibiting improved crashworthiness are sandwich structures [10,11]. The toucan beak is a representative example of a sandwich composite structure consisting of superposed hexagonal scales of keratin attached to each other and a fibrous network of closed cells comprising calcium-rich proteins. Sandwich structures inspired by coconut shells have a total of three layers, known as exocarp, mesocarp, and endocarp layers from the outside to the inside [12]. The structure of coconut shells can be imitated in the design of laminated structures, with a hard outer layer for penetration resistance and a soft inner layer for increased toughness and energy absorption. Various sandwich structures with prismatic lattices of triangular, trapezoidal, or rectangular cross-sections inserted into the voids of sandwich panels demonstrating high projectile-penetration resistance have been designed for various impulsive loading [13,14,15] and underwater cavitation [16] applications.

Flexible structures with fish-scale morphologies consisting of a bony layer and a collagen layer comprise another category of biomimetic structures. Bony layers are stiff, hard, and brittle due to their high mineral content, whereas the underlying collagen cross-ply is softer and more deformable [17]. Fish-scale-like structures generally show high penetration resistance and flexibility [18]. For example, carp scales have a lamellar collagen fiber structure for protection, while retaining the flexibility and maneuverability of the fish, with fiber stretching, rotation, and sliding mechanisms acting synergistically to delocalize damage [19]. Closed-cell structures imitating sea urchins fabricated by a material extrusion process that does not require support structures demonstrated an important cell-size effect on the compressive load-bearing capacity [20,21]. Enhanced perforation resistance without a loss of flexibility obtained by covering a soft substrate with a biomimetic carbon fiber-reinforced polymer composite with a microstructure inspired by fish scales revealed the high potential of flexible fish-scale structures for body armor applications [22]. The inherent strain-stiffening characteristics of a fish-scale structure were tuned by adjusting certain structural features, such as increasing the scale density or decreasing the scale–dermis rotational stiffness relative to the bending stiffness of the scale [23].

Contrary to the aforementioned classifications of biomimetic materials, cellular materials are arrays of spatial unit cells with edges and faces exhibiting unique mechanical properties. For instance, the hybrid edge- and vertex-based hierarchical arrangement of square honeycombs has been found to demonstrate a superior out-of-plane crushing performance compared with regular square honeycomb and edge-based hierarchical square honeycomb designs with the same mass [24]. The majority of lightweight cellular structures possess 3D lattice architectures consisting of hollow, thin-walled unit cells inspired by honeycomb designs, polyhedral plant cells [25], and the bamboo tube wall [26,27,28,29]. The tensile strength-to-specific weight ratio of bamboo is about six times greater than that of steel. The most common failure mechanisms of bamboo under tensile and compressive loading are fiber debonding and fiber splitting, respectively. In situ imaging nanoindentation studies of the cell wall mechanical properties of bamboo fibers and parenchyma cells revealed different deformation mechanisms of the cell walls when indented in the longitudinal and transverse direction of the bamboo fibers [30]. Due to the gradient distribution of the vascular bundles along the thickness direction, bamboo demonstrates anisotropic fatigue behavior. The unique strength, ductility, hardness, impact resistance, and fracture toughness of bamboo are attributed to the composite structure of numerous fibers consisting of cellulose microfibrils embedded in a matrix of intertwined hemicellulose and lignin. The bamboo cell wall possesses a microhierarchical structure comprising layers of unit cells distributed around individual unit cell walls, which provide a resistance to impact loading [31,32].

Bamboo is characterized by a functionally graded honeycomb structure with different honeycomb sizes, shapes, and wall thicknesses. Each hexagon is connected to its neighbors by a series of smaller square hollow structures (hereafter referred to as voids). As the hexagonal unit cells are hollow, the structure is much lighter than traditional solid hexagon structures. Remarkably, bamboo-like structures demonstrate four times higher shock absorption energy than traditional honeycomb structures [31,33]. The energy absorption capacity and crushing resistance of bamboo-inspired tubular honeycombs have been reported to correlate with the topology characteristics and structural hierarchy [34]. These exceptional attributes of bamboo have inspired the design of various bamboo-like structures. For example, a bamboo-inspired design approach was developed to fabricate mechanically flexible and electrically conductive carbon nanofiber/polydimethylsiloxane foam composites with unique hierarchical pore structures [35]. A simple and efficient two-step method has been invented to convert natural bamboo into a lightweight structural material exhibiting a superior strength and toughness due to its bamboo-like gradient laminated structure [36]. Statistical analysis and three-point bending tests of sandwich panels consisting of a bamboo core with different dimensions, packing geometry (cubic or hexagonal), and facing materials (aluminum or glass fiber-reinforced polymers) showed a dependence of the mechanical behavior on the shear strength and elastic modulus of the bamboo core and the flexural strength of the panels [37]. An appraisal of the mechanical properties and failure modes of laminated bamboo lumber has indicated that this material is a promising alternative to conventional building materials [38]. The foregoing studies suggest that many lightweight, bamboo-like structures with a wide range of hierarchical architectures and mechanical characteristics comparable with (or even superior to) those of counterpart engineering materials demonstrate a high potential to replace structural components in various industry sectors, such as construction, scaffolding, automotive, and aerospace. An important advantage of bamboo-inspired structures is that they are regular and repetitive; therefore, their design can be easily optimized and fabrication by 3D printing is facile. Furthermore, contrary to sandwich and fish-scale structures, the fabrication of bamboo structures does not require multiple composite materials.

Despite significant advances in bamboo fiber-reinforced composites [39,40,41], relatively less effort has been devoted to the development of cellular structures with lattices resembling those of the bamboo microstructure. Consequently, the objective of this study was to investigate the lattice dimension effects on the mechanical behavior of bamboo-like structures fabricated by stereolithography 3D printing. The mechanical properties of bamboo-like structures consisting of hollow honeycomb unit cells with varying sizes were extracted from the true stress–strain responses. High-speed camera video recordings were used to track the initiation and evolution of fracture-induced failures during transversal compressive loading. Numerical results obtained with the finite element method (FEM) yielded an insight into the failure patterns. The results of this study confirmed the strong dependence of the mechanical behavior of biomimetic structures on the unit cell size and number density.

## 2. Methods

### 2.1. Materials

A standard resin containing 55–75% urethane dimethacrylate, 15–25% methacrylate monomer, and <0.9% photo-initiator was used to fabricate the test structures. The resin possessed a 1.08 g/cm^3^ mass density, 1.6 GPa elastic modulus, 38 MPa ultimate tensile strength, and 12% elongation at fracture. When stereolithography resins are exposed to certain light wavelengths, short molecular chains join together, polymerizing monomers and oligomers into solidified rigid or flexible geometries.

### 2.2. Design of Test Structures

**Figure 1:**
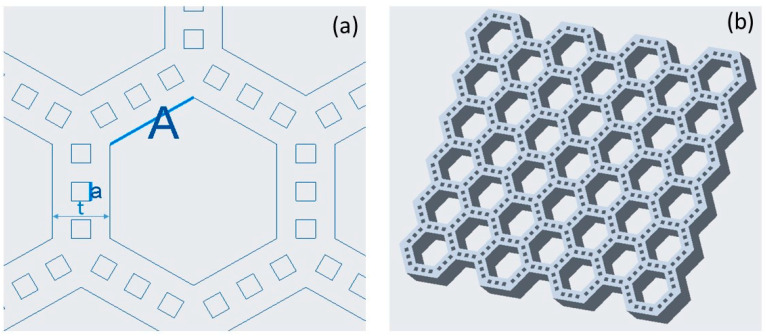
(**a**) A characteristic unit cell with labeled key design parameters and (**b**) a bamboo-like test structure without the flat plates at the top and the bottom.

Bamboo-like structures with unit cells similar to those of the natural material were designed based on a parametric study of the key unit cell dimensions. The main design parameters were the side length of the inner hexagonal cells *A* and the size of the square voids *a*, which were uniformly distributed on each side of the hexagonal unit cell, as shown in Figure 1a. To reduce the design matrix, the number of the small voids *n* on each side of the hexagonal cells was set to be equal to 3 and the length ratio *A*/*a* was fixed at 5. In addition, the wall thickness *t* in each structure was set to be equal to 3*a*. To investigate the effect of the structure architecture on the mechanical performance, five groups of unit cells with *A*/*a* = 2.5/0.5, 5/1, 7.5/1.5, 10/2, and 15/3 mm/mm were fabricated and tested. Hereafter, these structures are designated as A2.5, A5, A7.5, A10, and A15, for brevity. Figure 1b shows the design of a medium-size bamboo-like hexagonal structure with three small rectangular voids on each side of the unit cells. The overall size of the test structures was 100 × 110 × 10 mm. For most structures, 10-mm-thick plates were added at the top and the bottom to aid the uniform distribution of the compressive load applied during testing.

### 2.3. Fabrication of Test Structures

The test structures were fabricated by stereolithography 3D printing. The printing layer thickness was fixed at 0.1 mm. Extra support structures were used to ensure the integrity and viability of the printed structures and their facile removal from the printing platform. The whole printing process of each structure lasted for ~8 h. After printing, the structures were washed for 20 min and the printed parts were cleaned to remove the liquid resins from their surface and internal structure. Post-curing with light and heat is a key process step in this printing process; therefore, the structures were cured at 60 °C for 30 min. The smallest unit cell structures were cured at a lower temperature and for a longer time to prevent warping. The final step involved the removal of the support structure.

### 2.4. Mechanical Testing

**Figure 2:**
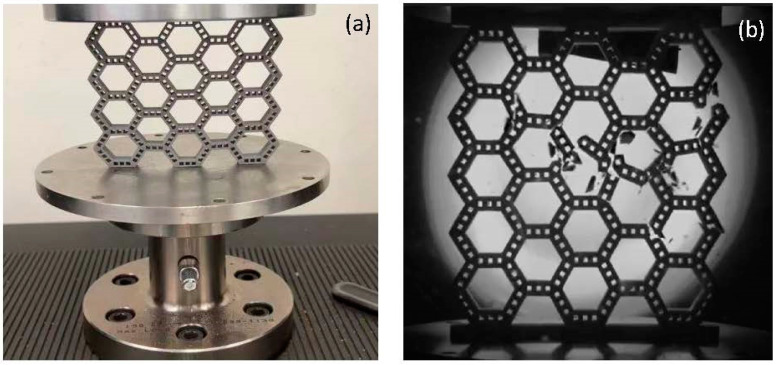
(**a**) A 4 × 4 unit cell test structure affixed between the rigid plates of the Instron machine and (**b**) fracture of a 5 × 5 unit cell test structure under transversal compressive loading captured by a high-speed camera.

Mechanical testing was performed with an Instron machine under quasistatic transversal compressive loading. The purpose for this type of testing was to determine the mechanical behavior of the bamboo-like structures without the added complexity of strain rate effects and/or rapid damage. Therefore, the loading rate was set at 2 mm/min. A high-speed camera was used to record the fracture process of each structure. Figure 2a shows a 4 × 4 unit cell test structure affixed between the rigid plates of the Instron machine. The recorded deformation of each structure was analyzed to obtain an insight into the instigation and progression of the failure process. The loading and deformation data were continuously acquired and recorded during the testing. Figure 2b shows a 5 × 5 unit cell test structure at the instant of a fracture-induced failure under transversal compressive loading. To evaluate the repeatability of the stress–strain responses and recorded failure evolution, 2–3 tests were performed with each structure. Tests that resulted in out-of-plane buckling due to the slenderness of the structures were discarded.

### 2.5. Finite Element Analysis

The general-purpose FEM code ANSYS was used to track the development of stresses and strains in the test structures throughout the entire test. The material properties obtained from the compression tests of solid specimens consisting of the same material were used as the input in the FEM model of the bamboo-like structures. The same uniform downward (compressive) displacement as in the experiments was quasistatically applied to all the nodes of one of the plates, whereas the nodes of the other plate were fully constrained to reproduce the boundary conditions in the mechanical tests. Large deformation was used in all simulations. The purpose of the FEM analysis was to determine the locations of maximum stress and strain in each structure as a function of the quasistatically applied compressive displacement, aiding the explanation of the failure of each structure. Additionally, the simulations provided animations of the deformation process, which were compared with those captured by the high-speed camera to verify the validity of the FEM model. For structures A7.5, A10, and A15, the size of the FEM mesh was the same as that of the structures; however, for structures A2.5 and A5, only one-half of the actual size of each structure was modelled to reduce the computational time and the horizontal movement of the nodes on the symmetry line was constrained for consistency with the symmetry of deformation in the experiments.

## 3. Results and Discussion

**Figure 3:**
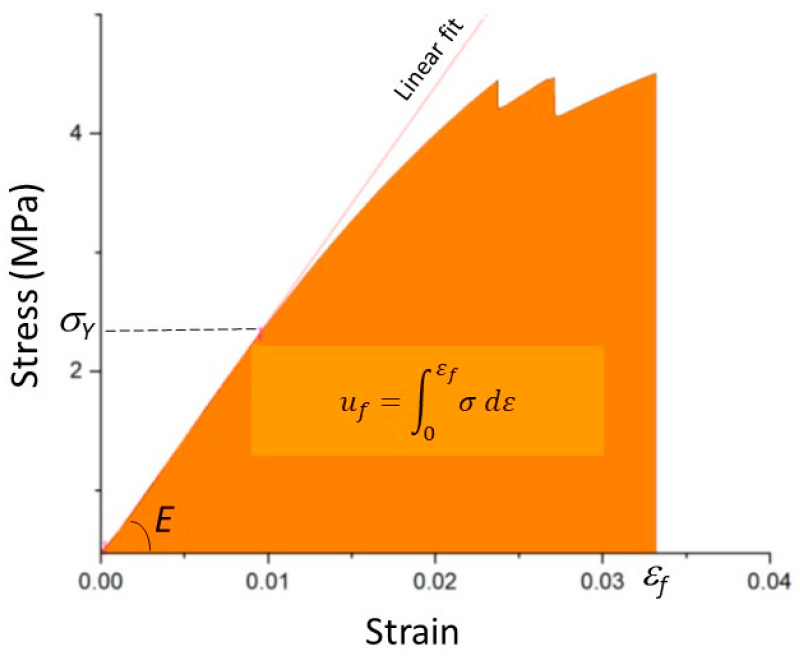
Representative true stress–strain response of structure A5 showing the calculation of the elastic modulus *E*, yield strength *σ_Y_*, strain at fracture *ε_f_*, and strain energy density at fracture *u_f_*.

True stress–strain responses derived from the force–displacement data recorded during testing were used to extract the critical mechanical properties of each structure. Figure 3 displays a representative stress–strain response, including characteristic material properties, such as the elastic modulus *E*, yield strength *σ_Y_*, strain at fracture *ε_f_*, and strain energy density at fracture *u_f_*. The elastic modulus was obtained by a linear fit through the stress–strain data of the elastic deformation range. The same method was used to calculate the elastic modulus from the FEM-simulated stress–strain response of each structure. The strain energy density was obtained as the area under the entire stress–strain response up to a specified strain (e.g., fracture strain *ε_f_*). The stress drops in the stress–strain response shown in Figure 3 before the commencement of the massive failure were due to microfracture events, detected in the video recording of the high-speed camera.

**Figure 4:**
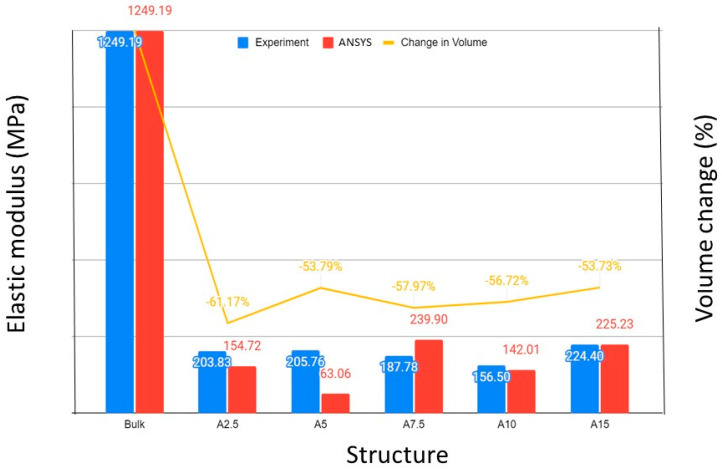
Experimental and FEM results of the elastic modulus of various bamboo-like structures. The elastic modulus of a solid (bulk) structure with the same dimensions and the volume change for each structure relative to the bulk structure are also included to illustrate the enhanced compliance and significantly lower weight of the bamboo-like structures.

The experimental results of the elastic modulus were contrasted with the simulation results to confirm the validity of the FEM model. Figure 4 shows a fair agreement between the experimental and simulation results for structures A2.5, A7.5, A10, and A15. The discrepancy between the results for structure A5 could be attributed to the mesh coarseness. The comparison validated the modeling assumptions and discretization scheme. Figure 4 also shows the volume change (with respect to a solid (bulk) structure with the same dimensions) for each structure. Both the experimental and simulation results indicated that the bamboo-like structures were significantly more compliant and lighter than the bulk structure. From elastic stiffness and weight perspectives, A10 appeared to be the most compliant and lighter structure. Thus, from the lowest compliance standpoint, the critical unit cell and void sizes are predicted to be about 10 and 2 mm, respectively. The results shown in Figure 4 illuminate a size-dependent mechanical behavior and also demonstrate how biomimicry can be used to engineer the compliance-to-weight ratio of bioinspired engineering materials.

**Figure 5:**
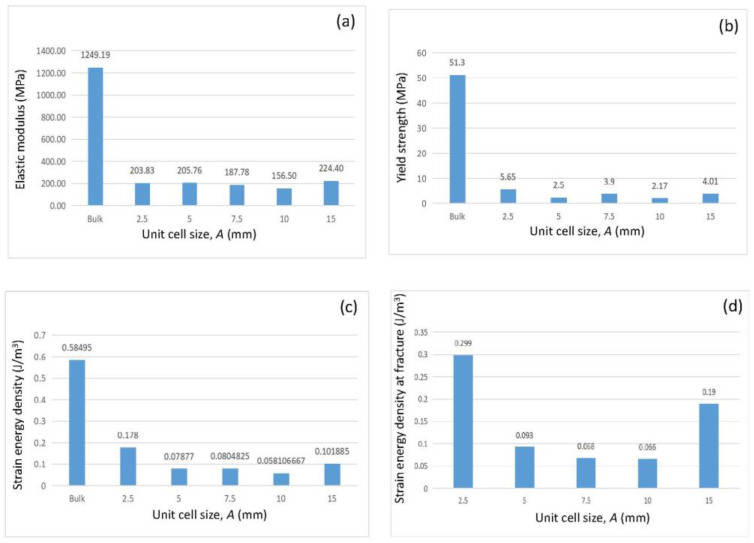
Mechanical properties of various bamboo-like structures: (**a**) elastic modulus, (**b**) yield strength, (**c**) strain energy density at 0.028 strain, and (**d**) strain energy density at fracture. Results for a solid (bulk) structure with the same dimensions and composition are also included for comparison.

Figure 5 shows the effect of the unit cell size on the mechanical characteristics of the bamboo-like structures. The mechanical properties of the bulk material are also included for comparison. Because the structures failed at different fracture strains, the results of the strain energy density at a fixed strain (i.e., 0.028) are also included for comparison. The results indicated that structures A2.5 and A15 exhibited the highest elastic modulus, yield strength, strain energy density, and fracture strength. However, if the change in volume (weight) was also taken into consideration, A2.5 appeared to be the preferred structure among all tested structures. Thus, the critical unit cell and void sizes for high strength and toughness bamboo-like structures were estimated to be about 2.5 and 0.5 mm, respectively. The elastic modulus, yield strength, fracture strength, and strain energy at fracture (obtained as the area under the stress–strain response) versus the unit cell size and void size of the tested structures are given in Table 1.

**Figure 6:**
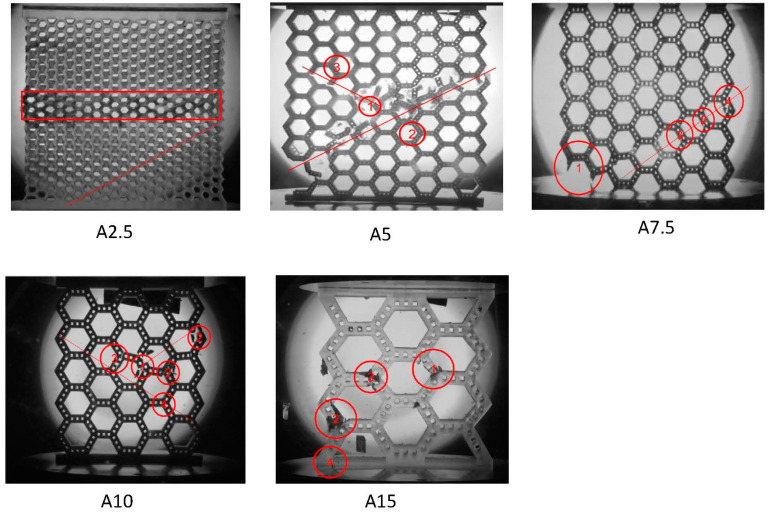
Evolution of the fracture process in the bamboo-like structures A2.5, A5, A7.5, A10, and A15. (The circled numbers correspond to the order of the fracturing events. The solid lines indicate the final fracture path at the instant of failure.).

Further insight into the deformation behavior of the bamboo-like structures was obtained from the video recordings. Selected images of the failed structures are presented to provide insight into the failure process. Figure 6 shows characteristic images of the failed structures. The circled regions denote the fracture points, the circled numbers indicate the sequence of the fracture events, and the lines represent the final fracture path. Due to the small unit cells of structure A2.5, it was difficult to discern the failure process from the images of this structure. Nevertheless, the distortion of the unit cells revealed a failure path at a slope of ~30°. Moreover, due to the small thickness of the structure, localized out-of-plane bending of the middle sections of this structure (enclosed by a red square) was also detected at the instant of failure. Although cracking in structure A5 was instigated at the bottom left corner, presumably due to localized stress concentration, this crack had no obvious and direct implication on the final fracture that commenced in the central region of the structure. The fracture in structure A5 was found to be a multistep process. A microcrack formed first in the central region of this structure, specifically at the highly stressed corner of a unit cell and, subsequently, propagated along a ~30° sloped path almost instantaneously in both directions. Multiple fracturing events were observed as small downward excursions in the stress–strain response (Figure 3). Finally, a large stress excursion occurred in the stress–strain response at the instant of failure by fracture.

Fracture initiation was first encountered at the left corner of structure A7.5 and was associated with stress concentration effects attributed to slight plate non-flatness and/or specimen misalignment. This fracture event was correlated with a small stress drop in the stress–strain response. Nevertheless, this experiment subsequently revealed a fracture process that resembled the fracture of other structures (i.e., a fracture path at a slope of ~30°). Divergent from the foregoing structures, the fracture in the A10 structure commenced almost instantaneously. From a frame-by-frame examination of the latest stage of deformation just before the abrupt failure, it was found that the first microfracture event was instigated at the center of this structure, consistent with the FEM predictions. At that juncture, the fracture rapidly propagated towards the edges of the structure, forming a fracture line with a slope of ~30°. Although the fracture angle was the same in all experiments with this structure (i.e., ~30°), the propagation of the fracture occurred towards either the left or the right edge of the structure, obviously depending on the presence of fabrication defects. The fracture in structure A15 was also initiated by a microcrack at the center of this structure, evinced in the stress–strain response as a small stress drop. This was followed by a fracture event at the left side of the structure, accompanied by a large stress drop. All tests of this structure showed a similar failure process. Due to the significantly fewer and larger unit cells in structure A15, a fracture line was not encountered at the instant of massive failure.

**Figure 7:**
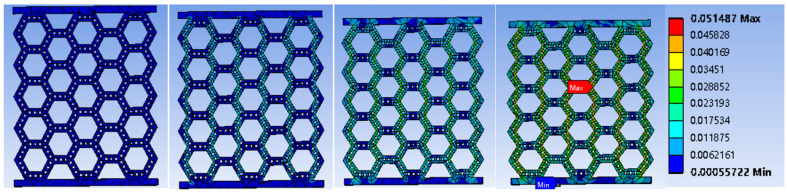
Representative strain maps in structure A10 at different deformation stages. (The compressive load increases from left to right.).

The FEM simulations provided an insight into the failure process of bamboo-like structures. It was found that the maximum stresses and strains occurred in the central region of the structures. As the deformation evolved, the highly stressed points aligned in a certain direction, closely resembling the fracture lines of the failed structures. The FEM animations revealed that larger stresses and strains developed at the corners of the hexagonal unit cells. A representative result of the progression of deformation in structure A10 is shown in Figure 7. The strain maps show that the highest strain eventually arose at the center of the structure (right panel), in qualitative agreement with the experimental observation of the crack initiation in the failed structures. Similar evidence was derived from the FEM simulations of other structures.

The present study provided an insight into the mechanical behavior of bamboo-inspired structures. From basic engineering and application perspectives, this study introduced a hybrid experimental-simulation methodology for the modification of the architecture of biomimetic structures to achieve an enhanced mechanical performance. Such an approach should be useful in creating innovative solutions that could be profitable in terms of structural applications, cost, and environmental compatibility.

## 4. Conclusions

Mechanical tests, high-speed video recording, and FEM simulations were used to examine the mechanical performance of bamboo-like structures fabricated by stereolithography 3D printing. The experimental and FEM results revealed a strong dependence of the mechanical behavior and weight of the bamboo-like structures on the dimensions and number density of the hexagonal unit cell. Most structures exhibited a similar failure process under transversal compressive loading, characterized by microfracture initiation within the central region of the structures, followed by rapid crack propagation along a sloped path of maximum strain. The obtained results demonstrated a dominant effect of the unit cell (lattice) dimensions on the mechanical behavior of bamboo-like structures.

The contribution of this study is the establishment of an experimental-simulation methodology for the tailoring of the architecture of biomimetic structures to achieve a specific mechanical performance, and the impetus to design biomimetic materials with specific microarchitectures, exploiting the structural advantages of the dependence of the mechanical behavior on the lattice characteristics. This investigation is part of an ongoing effort to develop physical structure-based mechanics approaches for the design of biomimetic structures with specific mechanical attributes through the establishment of functional relations between the architecture and the size, geometry, and 3D spatial distribution of the building blocks of biomimetic structures. Such a mechanical approach, coupled with efficient optimization schemes, may enable further advances in the development of new, strong, and sustainable materials, representing worthy alternatives to traditional engineering materials.

## Figures and Tables

**Table 1:** Mechanical properties of test structures with *n* = 3 and *A*/*a* = 5.

Structure	Unit Cell Size *A* (mm)	Void Size *a* (mm)	Elastic Modulus *E* (MPa)	Yield Strength *σ_Y_* (MPa)	Fracture Strength *σ_f_* (MPa)	Strain Energy Density at Fracture *u_f_* (J/m^3^)
A2.5	2.5	0.5	203.88	5.65	8.16	0.299
A5	5	1.0	205.76	2.50	4.55	0.093
A7.5	7.5	1.5	187.78	3.90	6.44	0.068
A10	10	2.0	156.50	2.17	3.76	0.066
A15	15	3.0	224.40	4.01	6.91	0.190

## Data Availability

All data of this work are included in the published paper.
